# Programmed cell death evasion in BRAF V600E-driven primary CNS tumors and thyroid cancer brain metastases

**DOI:** 10.3389/fneur.2026.1921351

**Published:** 2026-09-02

**Authors:** Tong Jin, Yuanshi Lv, Qiushi Yang, Rui Zhang, Hui Jin

**Affiliations:** 1 Department of Thyroid and Head and Neck Surgery II, Jilin Provincial Cancer Hospital, Changchun, China; 2 Department of Thyroid and Head and Neck Surgery I, Jilin Provincial Cancer Hospital, Changchun, China; 3 Department of Internal Medicine III, Jilin Provincial Cancer Hospital, Changchun, China

**Keywords:** apoptosis, blood–tumor barrier, BRAF V600E, ferroptosis, ganglioglioma, pleomorphic xanthoastrocytoma, thyroid cancer brain metastases

## Abstract

BRAF V600E occurs in selected primary central nervous system (CNS) tumors and in a subset of thyroid cancers that can metastasize to the brain, but the shared alteration does not make these diseases biologically equivalent. This Mini Review examines programmed cell death evasion in pleomorphic xanthoastrocytoma and ganglioglioma, with selected molecular and clinical comparisons involving epithelioid glioblastoma, pilocytic astrocytoma, pediatric low-grade glioma, and thyroid cancer brain metastases (TCBM). Evidence linking BRAF–MEK–ERK signaling to apoptosis is strongest in non-CNS thyroid cancer and other BRAF-mutant models; direct validation in TCBM remains limited. Ferroptosis sensitization has been demonstrated preclinically in BRAF V600E anaplastic thyroid cancer outside the brain, whereas the ferroptosis suppressor protein 1 (FSP1)/coenzyme Q10 (CoQ10) axis and dihydroorotate dehydrogenase (DHODH) remain general mechanistic frameworks rather than established dependencies in the target CNS settings. Clinical studies support BRAF/MEK inhibition in BRAF V600E-mutant glioma and systemic anaplastic thyroid cancer, but direct TCBM intracranial evidence is restricted to very limited, confounded observations. The intact blood–brain barrier, heterogeneous blood–tumor barrier, efflux transporters, and brain microenvironment further complicate inference from systemic outcomes. We therefore separate direct, indirect, and hypothesis-generating evidence and identify disease-specific pharmacokinetic, mechanistic, and prospective clinical studies as priorities.

## 1 Introduction

Primary CNS tumors and brain metastases are separated by lineage, histology, natural history, and treatment context. This review focuses on selected tumors in which BRAF V600E is clinically or biologically relevant: pleomorphic xanthoastrocytoma (PXA) and ganglioglioma as the principal primary CNS comparators; epithelioid glioblastoma, pilocytic astrocytoma, and pediatric low-grade glioma (pLGG) for molecular or clinical context; and thyroid cancer brain metastases (TCBM), with papillary thyroid carcinoma (PTC), poorly differentiated thyroid carcinoma (PDTC), and anaplastic thyroid carcinoma (ATC) evidence used only when its extracranial origin is explicit. The clinical importance of TCBM is supported by a 27-patient cohort in which median survival after CNS-metastasis diagnosis was 5.0 months, although histologies and treatments were heterogeneous and outcomes were not stratified by BRAF status (1).

BRAF V600E denotes the replacement of valine by glutamic acid at codon 600. Constitutive mitogen-activated protein kinase (MAPK) pathway signaling provides a rationale for BRAF inhibitor (BRAFi) and MEK inhibitor (MEKi) therapy, but a shared driver is not a shared disease identity. The relative importance of apoptosis, ferroptosis, lineage-specific co-alterations, immune context, and CNS drug exposure differs across the included entities. Accordingly, the comparison is used as a structured framework for asking which mechanisms are established in a stated disease context, which are indirect, and which remain untested. It is not used to infer that a mechanism demonstrated in melanoma, colorectal cancer, extracranial thyroid cancer, or another brain-metastasis model is established in PXA, ganglioglioma, or TCBM.

**Figure 1:**
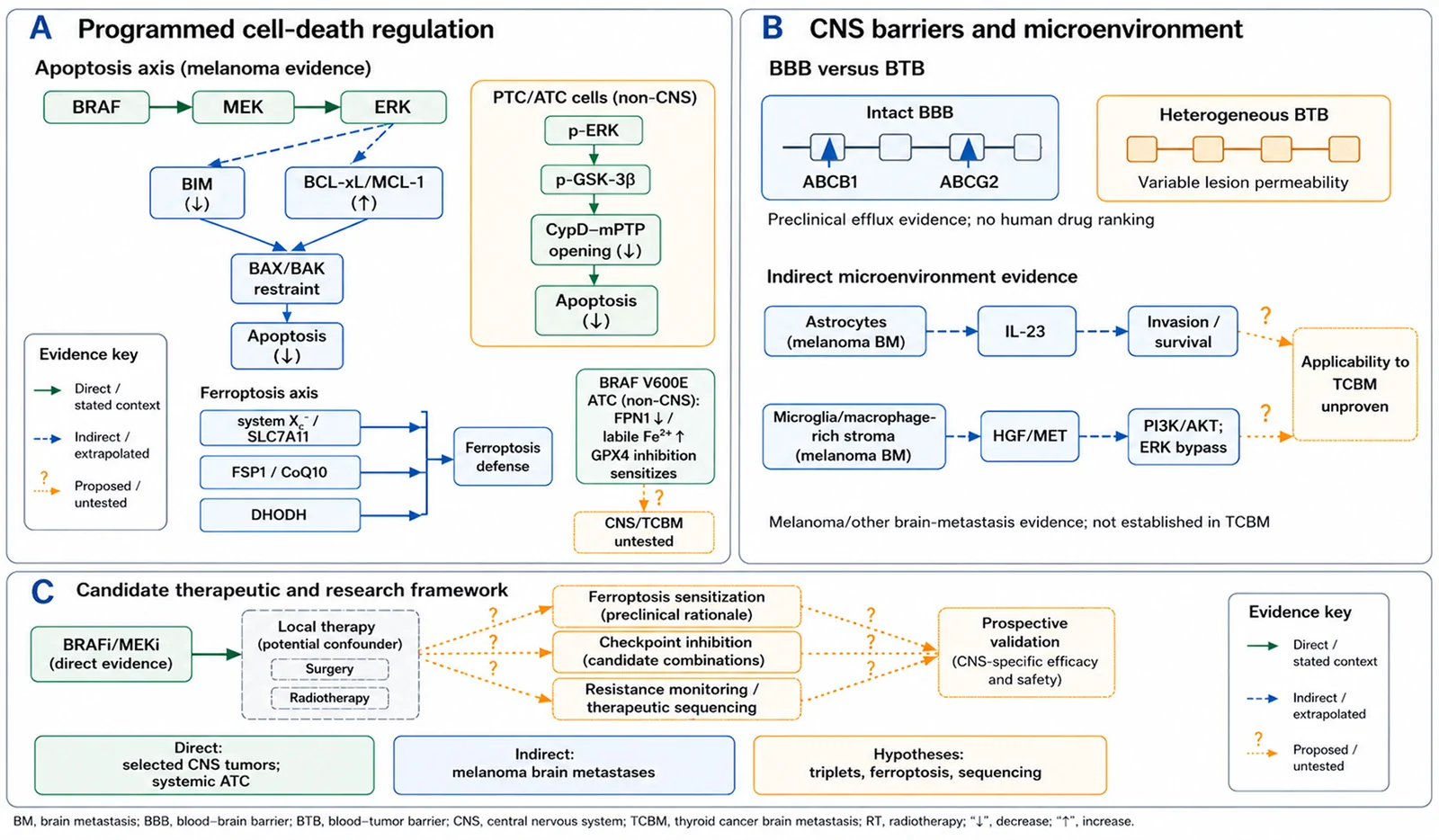
Evidence-stratified framework for programmed cell-death regulation, CNS barriers, and therapeutic hypotheses in selected BRAF V600E-associated primary CNS tumors and thyroid cancer brain metastases. Panel **(A)** separates melanoma-derived apoptosis evidence, non-CNS thyroid-cell ERK/GSK-3β/CypD/mPTP evidence, general ferroptosis defenses, and BRAF V600E anaplastic-thyroid-cancer ferroptosis findings (9–16). Panel **(B)** distinguishes the intact blood–brain barrier (BBB) from the heterogeneous blood–tumor barrier (BTB), depicts preclinical ABCB1/ABCG2 efflux, and labels reactive-astrocyte/IL-23 and stromal HGF/MET bypass evidence as indirect brain-metastasis evidence (17–24). Panel **(C)** separates directly supported BRAF inhibitor (BRAFi)/MEK inhibitor (MEKi) treatment contexts from indirect melanoma comparators and proposed combination strategies. Solid arrows denote directly demonstrated relationships in the explicitly stated context; dashed arrows denote indirect or extrapolated evidence; dotted arrows with a question mark denote proposed or hypothesis-generating relationships. This figure is original.

Programmed cell death is considered here through two tractable axes: mitochondrial apoptosis and ferroptosis. Their relevance is linked to clinical activity of MAPK inhibition, yet clinical response alone cannot identify the death pathway engaged in a patient lesion. The review therefore keeps mechanistic studies separate from treatment-outcome studies and treats the blood–brain barrier (BBB), blood–tumor barrier (BTB), and brain microenvironment as modifiers rather than deterministic explanations for resistance. The evidence-stratified framework used throughout this review is summarized in Figure 1.

## 2 Narrative literature search and evidence classification

The literature search included publications available through July 29, 2026. PubMed and PubMed Central records, Crossref metadata, journal and DOI pages, and primary trial publications were examined using combinations of terms for BRAF V600E, PXA, ganglioglioma, epithelioid glioblastoma, pilocytic astrocytoma, pLGG, thyroid cancer, TCBM, apoptosis, BIM, BCL-xL, MCL-1, ferroptosis, SLC7A11, GPX4, FSP1, DHODH, BBB, BTB, ABCB1, ABCG2, astrocytes, and immunotherapy. Searches also targeted named trials and regimens when numerical clinical outcomes required verification.

Primary peer-reviewed experimental studies and original clinical-trial reports were prioritized. Reviews were used for orientation or cross-checking but not as substitutes for primary support for clinical outcomes. Search-result snippets, news reports, company pages, and secondary medical websites were not used to confirm response rates, survival, frequencies, or pharmacokinetic parameters. Preprints were checked for a formally published version; the peer-reviewed publication was used when available. The republished 2023 TRICOTEL report was used instead of the retracted 2022 version.

Evidence was classified as direct clinical or preclinical evidence in the stated target CNS context, evidence in the same tumor type outside the CNS, indirect evidence from another brain-tumor or brain-metastasis context, evidence from another BRAF-mutant cancer, general pathway biology, or hypothesis-generating extrapolation. Numerical data were retained only when traceable to an audited primary source; unavailable outcomes are reported as not reported (NR). This was a narrative review and did not use a PRISMA workflow, protocol registration, dual independent screening, formal risk-of-bias assessment, or meta-analysis.

## 3 Molecular landscape of selected BRAF V600E-associated CNS tumors and thyroid cancer brain metastases

BRAF V600E frequency is highly dependent on histology, referral population, assay, anatomic site, and classification era. In a direct-sequencing series, the alteration was detected in 42 of 64 grade 2 PXA (66%) and 15 of 23 tumors then classified as anaplastic PXA (65%). A second selected PXA cohort using VE1 immunohistochemistry with sequencing identified BRAF V600E in 38 of 49 tumors (78%) (2, 3). These estimates should not be treated as universal prevalence estimates because cohort selection and contemporary classification affect the denominator. In the same historical direct-sequencing study, BRAF V600E occurred in 14 of 77 grade 1 gangliogliomas (18%) and 3 of 6 anaplastic gangliogliomas; the six-case denominator and historical category limit interpretation (2).

The mutation is less uniformly distributed in pilocytic astrocytoma. Schindler et al. (2) detected it in 9 of 97 tumors (9%), including 4 of 12 diencephalic tumors, emphasizing site dependence and the need to distinguish BRAF V600E from BRAF fusions. In a pediatric low-grade glioma cohort, 69 of 405 evaluable tumors (17%) carried BRAF V600E, but proportions varied markedly by histology: 5 of 167 pilocytic astrocytomas, 25 of 51 gangliogliomas, and 7 of 9 PXA (4). A small historical epithelioid glioblastoma series identified the mutation in 7 of 13 tumors (54%) by mutational analysis; this estimate is classification-sensitive because some historical tumors may be reassigned under contemporary integrated diagnosis (5).

Thyroid-cancer estimates require the same contextual discipline. Lan et al. (6) analyzed a selected 66-patient PTC cohort comprising 20 patients with distant metastasis and 46 patients without distant metastasis. BRAF V600E was identified in 48 of 66 tumors (72.7%) overall. This cohort-wide estimate is neither a prevalence estimate for metastatic PTC nor a general prevalence estimate for classical PTC. A separate single-institution cohort reported the alteration in 162 of 185 classical PTCs (88%), with strong dependence on subtype and testing context (7). These values must not be combined into a universal frequency range. In recurrent PTC, coexisting BRAF V600E and TERT-promoter mutations were strongly associated with loss of radioiodine avidity. This association does not establish deterministic progression to ATC or brain metastasis (8). Available TCBM cohorts do not provide a reliable BRAF-stratified frequency or uniform molecular profile. Table 1 therefore presents cohort-bound estimates and retains NR where an audited primary source did not report a required item.

**Table 1:** Molecular landscape and clinical evidence in BRAF V600E-associated selected primary CNS tumors and thyroid cancer brain metastases.

Tumor/entity	Cohort and assay context	BRAF V600E frequency	Key molecular/clinical context	Evidence limitations	Reference
Part A. Molecular and clinicopathological landscape					
Pleomorphic xanthoastrocytoma (PXA)	Direct sequencing: 64 grade 2 and 23 historical anaplastic tumors; separate selected cohort used VE1 IHC plus sequencing.	42/64 (66%); 15/23 (65%); 38/49 (78%)	Enrichment in selected cohorts; temporal location and CD34/reticulin associations reported.	Referral selection and historical classification affect denominators; not a universal prevalence.	(2, 3)
Ganglioglioma	Historical direct-sequencing series; separate multi-institution pediatric low-grade glioma (pLGG) cohort.	14/77 grade 1 (18%); 3/6 historical anaplastic; 25/51 in pLGG cohort	Frequency varies by grade, age, site, and cohort composition.	Six-case anaplastic denominator and historical terminology limit generalization.	(2, 4)
Pilocytic astrocytoma	Direct-sequencing series and multi-institution pLGG cohort; BRAF V600E distinguished from BRAF fusions.	9/97 (9%); 4/12 diencephalic; 5/167 in pLGG cohort	Anatomic-site dependence is important.	Different assays, referral populations, and molecular classes; estimates are not interchangeable.	(2, 4)
Pediatric low-grade glioma	Multi-institution cohort; 405 tumors evaluable for BRAF V600E across several histologies.	69/405 (17%) overall	Marked histology-specific variation: PXA, ganglioglioma, and pilocytic astrocytoma differ.	Aggregate frequency should not be assigned to an individual histology.	(4)
Historical epithelioid glioblastoma	Small mutational-analysis series classified under the diagnostic framework of its era.	7/13 (54%)	BRAF V600E was enriched in this historical morphology.	Contemporary integrated diagnosis may reclassify some historical cases.	(5)
PTC cohort with and without distant metastasis	Selected 66-patient cohort: 20 with distant metastasis and 46 without; whole-exome sequencing and/or a 425-gene targeted panel.	48/66 (72.7%) in the complete selected cohort	The study compared genomic features associated with distant metastasis.	Not metastatic-PTC prevalence, general classical-PTC prevalence, or TCBM frequency.	(6)
Classical papillary thyroid carcinoma	Single-institution cohort: 301 PTCs overall, including 185 classical PTCs; VE1 IHC and/or molecular testing.	162/185 (88%) in the classical-PTC subset	A high, subtype- and testing-context-dependent estimate.	Single institution; cannot be combined with the Lan cohort into a universal range or used as TCBM frequency.	(7)
Thyroid cancer brain metastases (TCBM)	Clinical cohort of 27 patients with heterogeneous thyroid histologies, treatments, and CNS presentations.	NR in BRAF-stratified form	Median survival after CNS-metastasis diagnosis was 5.0 months.	No BRAF-stratified frequency or outcome; heterogeneous histology and treatment.	(1)
Tumor/context	Regimen, study design, and sample size	Overall/systemic response as reported	Intracranial response	PFS/OS	Key safety data	Evidence level and principal limitations	Reference
Part B. Clinical evidence for BRAF/MEK-targeted therapy							
Adult BRAF V600E glioma (ROAR)	Dabrafenib + trametinib; phase 2 basket; LGG *n* = 13, HGG *n* = 45	ORR: LGG 54%; HGG 33%	Primary CNS tumors; no separate intracranial endpoint	PFS: LGG 9.5 mo, HGG 5.5 mo; OS: LGG NE, HGG 17.6 mo	Any serious AE: LGG 3/13 (23%); HGG 16/45 (36%)	Direct CNS clinical evidence; heterogeneous histologies and single-arm design.	(25, 26)
BRAF V600-mutant glioma (VE-BASKET)	Vemurafenib; open-label basket subgroup; *n* = 24	ORR 25%	Primary CNS tumors; no separate intracranial endpoint	Median PFS 5.5 mo; OS NR here	Grade 3/4 maculopapular rash 3/24 (13%); AE discontinuation 1/24	Direct CNS clinical evidence; monotherapy and heterogeneous histologies.	(27)
Pediatric BRAF V600-mutant LGG	Dabrafenib + trametinib vs chemotherapy; randomized; 73 vs 37 (safety: 73 vs 33)	ORR 47% vs 11%	Primary CNS tumors; no separate intracranial endpoint	Median PFS 20.1 vs 7.4 mo; OS NR	Targeted arm: all-cause grade ≥3 AEs 34/73 (47%); treatment-related grade ≥3 19/73 (26%); AE discontinuation 3/73 (4%)	Direct randomized pediatric evidence; not generalizable to adult entities.	(28)
Pediatric BRAF V600-mutant LGG	Trametinib or dabrafenib + trametinib; early study; *n* = 13 and *n* = 36	ORR 15% and 25%	Primary CNS tumors; no separate intracranial endpoint	PFS/OS NR	AE discontinuation: trametinib 7/13 (54%); combination 8/36 (22%)	Direct pediatric clinical evidence; nonrandomized cohorts.	(29)
BRAF V600E anaplastic thyroid carcinoma	Dabrafenib + trametinib; ROAR phase 2 basket cohort; *n* = 36	Systemic ORR 56%; median response duration 14.4 mo	No brain-metastasis subgroup or intracranial endpoint reported	Median PFS 6.7 mo; OS 14.5 mo	Any serious AE 20/36 (56%); none of the trial's fatal serious AEs was treatment related	Direct systemic ATC evidence only; not TCBM intracranial evidence.	(26)
BRAF V600E thyroid cancer; two baseline TCBM cases	Dabrafenib + trametinib; single-center retrospective; *n* = 16 (10 PTC, 6 ATC)	Systemic ORR: PTC 50%; ATC 67%	One evaluable case: 39.7% intracranial reduction after prior SRT; no cohort iORR	PTC PFS/OS NE; ATC median PFS 2.7 mo, OS 3.6 mo	All-cause grade ≥3 AEs 6/16 (38%); one treatment-related death from interstitial pneumonitis	Direct but very limited TCBM observation; prior SRT and *n* = 2 baseline cases confound attribution.	(30)
Melanoma brain metastases (COMBI-MB)	Dabrafenib + trametinib; multicohort phase 2; *n* = 125 (cohort A *n* = 76)	Overall response not used as a TCBM endpoint	Cohort A iORR 58%	Cohort A median PFS 5.6 mo; OS 10.8 mo	All-cause grade 3/4 AEs 60/125 (48%); AE discontinuation 12/125 (10%)	Indirect melanoma comparator; cannot establish TCBM efficacy or drug penetration.	(31)
Melanoma brain metastases (BREAK-MB)	Dabrafenib; multicenter phase 2; *n* = 172	Overall response not used as a TCBM endpoint	BRAF V600E iORR: 39.2% without and 30.8% after prior local therapy	PFS/OS NR here	NR (no specific value retained without primary-report verification)	Indirect melanoma comparator; cohorts differed by prior local therapy.	(32)
Symptomatic melanoma brain metastases	Vemurafenib; open-label pilot; *n* = 24 evaluable; *n* = 19 measurable intracranial	10/24 overall partial responses involving intracranial and extracranial sites	7/19 had >30% intracranial regression; 3/19 confirmed intracranial partial responses	Median PFS 3.9 mo; OS 5.3 mo	Grade 3 AEs 4/24 (17%)	Indirect, small, symptomatic, previously treated melanoma cohort; endpoints have different denominators.	(33)
Melanoma CNS metastases (TRICOTEL)	Atezolizumab + vemurafenib + cobimetinib; republished phase 2; 65 enrolled, 60 treated	Overall response not used as a TCBM endpoint	Independent-review iORR 42%; intracranial response duration 7.4 mo	PFS/OS not used here	Treatment-related grade ≥3 AEs 41/60 (68%); one treatment-related death (limbic encephalitis)	Indirect melanoma comparator; valid 2023 republication, not the retracted 2022 article.	(37)

AE, adverse event; ATC, anaplastic thyroid carcinoma; BBB, blood–brain barrier; BTB, blood–tumor barrier; CNS, central nervous system; HGG, high-grade glioma; IHC, immunohistochemistry; iORR, intracranial objective response rate; LGG, low-grade glioma; mo, months; NE, not estimable; NR, not reported or not separately reported; ORR, objective response rate; OS, overall survival; PFS, progression-free survival; PTC, papillary thyroid carcinoma; PXA, pleomorphic xanthoastrocytoma; SRT, stereotactic radiotherapy; TCBM, thyroid cancer brain metastases. Values are study-specific and were not pooled. Safety definitions are stated explicitly. Melanoma brain-metastasis studies are indirect comparators and cannot establish efficacy in TCBM or primary BRAF V600E-mutant CNS tumors.

This distribution has two implications for the comparison. First, molecular eligibility must be confirmed within each histologic entity rather than inferred from a broad diagnostic label. Second, frequency is not a measure of pathway dependence, cell-death phenotype, or treatment benefit. A high proportion in a selected PXA or classical PTC cohort does not show that the two diseases share co-alterations or a response mechanism. Conversely, a lower frequency in ganglioglioma or pilocytic astrocytoma does not reduce the potential relevance of a confirmed mutation for an individual patient. These distinctions are especially important for TCBM, where the brain-metastasis cohort evidence is sparse and does not permit reconstruction of a BRAF V600E prevalence estimate from extracranial PTC cohorts.

## 4 Programmed cell death evasion

MAPK activation can alter the threshold for cell death, but the evidence is uneven across the reviewed tumors. Mechanistic components should be interpreted at the level of the experimental model in which they were demonstrated. An integrated BRAF–apoptosis–ferroptosis network has not been directly established in TCBM, and genotype-defined ferroptosis studies in the selected primary CNS tumors remain lacking.

### 4.1 Apoptosis-related mechanisms

Mitochondrial apoptosis is governed by the balance between BH3-only proteins, anti-apoptotic BCL-2 family members, and the BAX/BAK effectors that enable mitochondrial outer-membrane permeabilization (MOMP). In BRAF V600E melanoma models, ERK-dependent signaling reduced the pro-apoptotic protein BIM through phosphorylation-linked turnover (9). Melanoma studies also implicated MCL-1 upregulation in resistance to BRAFi alone or with MEKi, while combined analyses of MCL-1 and BCL-xL showed how anti-apoptotic buffering can constrain BAX/BAK-dependent death during MAPK inhibition (10, 11). These experiments provide an indirect mechanistic framework; they do not demonstrate the same dependency in PXA, ganglioglioma, or TCBM.

More disease-proximal evidence comes from non-CNS thyroid-cancer models. In PTC and ATC cell systems, BRAF V600E-associated mitochondrial phospho-ERK/phospho-GSK-3β signaling was linked to cyclophilin D (CypD) regulation, inhibition of mitochondrial permeability transition pore (mPTP) opening, and reduced staurosporine-induced cell death (12). This supports a thyroid cancer-specific mitochondrial mechanism outside the brain, not a mechanism demonstrated in TCBM patient samples. It also should not be combined with melanoma BIM or MCL-1 findings to imply that a single complete apoptotic circuit has been proven across all BRAF V600E tumors.

For selected primary CNS tumors, clinical sensitivity to BRAFi/MEKi establishes MAPK dependence in some lesions but does not establish which apoptotic nodes mediate response. Direct studies testing BIM degradation, BCL-xL/MCL-1 stabilization, BAX/BAK activation, MOMP, and the mitochondrial ERK/GSK-3β/CypD/mPTP axis in molecularly defined PXA, ganglioglioma, and TCBM are needed. Until then, these nodes may contribute to resistance and provide experimentally testable hypotheses, but disease-specific causality remains to be validated.

A useful experimental sequence would therefore test pathway suppression, BIM abundance, anti-apoptotic buffering, BAX/BAK activation, and mitochondrial permeabilization within the same genotype-defined model. Rescue experiments would be needed to distinguish association from dependence. For TCBM, paired extracranial and intracranial material would also be required to determine whether the brain site changes the apoptotic threshold. Without such linked measurements, a radiographic response to BRAFi/MEKi should be described as clinical activity rather than proof that a particular apoptotic node was restored.

### 4.2 Ferroptosis-related mechanisms

Ferroptosis is driven by iron-dependent lipid peroxidation and opposed by several redox-defense systems. The system Xc–transporter, which includes SLC7A11, supports cystine uptake and glutathione (GSH) synthesis; glutathione peroxidase 4 (GPX4) reduces phospholipid hydroperoxides. Ferroptosis suppressor protein 1 (FSP1) can regenerate reduced coenzyme Q10 (CoQ10) at membranes independently of GPX4, and mitochondrial dihydroorotate dehydrogenase (DHODH) provides a complementary defense in general cancer models (13, 14). FSP1/CoQ10 and DHODH are established general mechanisms, not validated dependencies in TCBM or the selected BRAF V600E primary CNS tumors.

The most relevant genotype-defined evidence is preclinical and extracranial. In BRAF V600E ATC models, the GPX4 inhibitors RSL3 and ML162 induced ferroptotic death, and dabrafenib plus RSL3 was synergistic; activity was also enhanced with a GPX4-inhibitor analog *in vivo* (15). Compound-specific results prevent broader claims. Erastin showed greater activity in BRAF V600E cells, but dabrafenib plus erastin was not synergistic. *In vivo*, dabrafenib plus imidazole ketone erastin (IKE) did not significantly improve on IKE alone. The same study associated BRAF V600E with reduced ferroportin-1, a larger labile iron pool, oxidative stress, and susceptibility to selected ferroptosis interventions. These observations do not establish efficacy in patients or in brain metastases.

Anlotinib reduced GPX4 and iron-homeostasis proteins and increased reactive oxygen species in ATC models; autophagy blockade potentiated its effects *in vitro* and *in vivo* (16). This BRAF-unselected, non-CNS ATC evidence can support a preclinical strategy but not a TCBM-specific therapy. RSL3, ML162, erastin, and IKE likewise remain experimental probes rather than established treatments for the target diseases. Studies of hepatocellular carcinoma, osteoarthritis, and broad ferroptosis biology were not used to claim target-disease specificity.

The priority is therefore not to assume a universal ferroptosis-resistant state, but to measure lipid-peroxidation sensitivity, iron handling, SLC7A11/GPX4 dependence, FSP1/CoQ10 activity, and DHODH dependence in molecularly characterized PXA, ganglioglioma, and paired thyroid primary/brain metastasis models. Such studies should test whether MAPK inhibition changes ferroptosis sensitivity in a compound- and context-dependent manner and should include CNS exposure and toxicity constraints.

Interpretation should also remain intervention-specific. Sensitivity to one GPX4 inhibitor cannot be used to predict sensitivity to system Xc–inhibition, and a genotype-associated difference for erastin does not establish synergy with dabrafenib. Likewise, an increased labile iron pool may create vulnerability but is not itself evidence that ferroptosis occurs in a treated patient. Minimum target-context studies should combine genetic and pharmacologic perturbation, verify lipid peroxidation-dependent death, and demonstrate rescue by a ferroptosis-specific intervention. In brain models, these endpoints should be interpreted alongside neuronal and glial toxicity and the unbound exposure achieved at the lesion.

## 5 CNS-specific barriers to therapeutic activity

### 5.1 Blood–brain barrier vs. blood–tumor barrier

The intact BBB and tumor-associated BTB are related but not interchangeable. The BBB is formed by specialized endothelial junctions and transport systems that regulate access to normal brain. Primary CNS tumors and brain metastases can disrupt this architecture, but disruption is spatially heterogeneous. In experimental brain metastases, permeability varied among and within lesions, and drug uptake could remain lower than in peripheral tissue despite contrast enhancement (17). Glioma infiltration beyond enhancing tumor and lesions altered by surgery or radiotherapy add further heterogeneity. Thus, neither an intact-BBB model nor the presence of a leaky tumor core represents the entire clinical exposure problem.

ABCB1 (P-glycoprotein) and ABCG2 (breast cancer resistance protein) can limit brain distribution of BRAF inhibitors in preclinical systems. Preclinical mouse studies show that ABCB1 and ABCG2 substantially restrict the total brain distribution of both dabrafenib and vemurafenib (18, 19). Because the studies used different dosing and sampling conditions, their absolute brain-to-plasma ratios should not be interpreted as a clinical head-to-head comparison or as human K_p, uu, brain_ values. These data do not establish that either agent has a validated exposure advantage in TCBM.

The unbound brain-to-plasma partition coefficient (K_p, uu, brain_) describes equilibrium partitioning of pharmacologically unbound drug between brain and plasma. It is a pharmacokinetic exposure metric, not a clinical response endpoint (20). Clinical intracranial activity integrates drug exposure, regimen potency, tumor biology, prior local treatment, and BTB heterogeneity. Accordingly, response rates from separate melanoma trials cannot be used to infer that one BRAFi crosses the BBB better than another. No audited evidence established clinically relevant saturation of ABCB1/ABCG2, superior encorafenib CNS penetration, or direct BRAF-inhibitor exposure and K_p, uu, brain_ data in TCBM.

These distinctions also affect clinical reporting. Contrast enhancement can identify regions of BTB disruption, but it does not quantify unbound drug at the target, and a response after local radiotherapy can reflect treatment interaction as well as systemic therapy. Pharmacokinetic studies should therefore specify the sampled compartment, total vs. unbound concentration, timing, and relationship to enhancing and infiltrative tumor. Until those data are available, CNS activity and brain penetration should be reported as separate questions.

### 5.2 Brain microenvironment and adaptive resistance

Reactive astrocytes and other stromal cells can either support or restrain brain tumors depending on context. In melanoma brain-metastasis models, tumor-conditioned astrocytes expressed interleukin-23 (IL-23), which enhanced invasion, and astrocyte-conditioned signaling activated PI3K/AKT and reduced vemurafenib sensitivity (21, 22). A separate melanoma brain-metastasis study implicated stromal hepatocyte growth factor (HGF)–MET signaling, with emphasis on microglia/macrophage-rich stroma rather than a universal astrocytic source (23). These data are indirect for thyroid-derived brain metastases.

In lung- and breast-cancer brain-metastasis models, tumor-expressed serpins protected metastatic cells from plasmin-mediated Fas ligand killing and supported vascular co-option (24). This provides another indirect framework, not a BRAF V600E-specific mechanism. Direct evidence for a reactive-astrocyte FGF2/FGFR pathway in TCBM was not established and is not asserted here. HGF/c-MET, IL-23, PI3K/AKT, RTK–ERK bypass, and serpin/plasmin pathways may therefore provide compensatory survival signaling, but each requires validation in the stated primary CNS tumor or TCBM context.

## 6 Clinical evidence for BRAF/MEK-targeted therapy

Clinical evidence must be separated by disease and by whether the measured lesion was intracranial. For primary CNS tumors, the tumor itself is located in the CNS, but trial reports may still use overall radiographic response rather than a separately defined intracranial endpoint. For thyroid cancer, systemic outcomes do not establish activity in brain metastases. Table 1, Part B therefore reports NR when a distinct endpoint was not available and does not combine trials.

Selected primary CNS tumors. In the phase 2 ROAR basket trial, dabrafenib plus trametinib produced an investigator-assessed objective response rate (ORR) of 54% among 13 patients with BRAF V600E low-grade glioma; median progression-free survival (PFS) was 9.5 months and overall survival (OS) was not estimable. Among 45 patients with high-grade glioma, ORR was 33%, median PFS 5.5 months, and median OS 17.6 months (25, 26). Histologic heterogeneity and the single-arm basket design limit subtype-specific inference. VE-BASKET evaluated vemurafenib monotherapy in 24 patients with BRAF V600-mutant glioma and reported ORR 25% and median PFS 5.5 months; heterogeneous histologies and monotherapy further limit comparison with combination trials (27).

Pediatric data provide stronger comparative evidence in a defined trial setting but should not be merged with adult cohorts. In a randomized study of 110 children with BRAF V600-mutant low-grade glioma, 73 received dabrafenib plus trametinib and 37 received chemotherapy. Independently assessed ORR was 47% vs. 11%, and median PFS was 20.1 vs. 7.4 months (28). An earlier study enrolled 49 patients: 13 received trametinib monotherapy and 36 received the combination. Independent-review ORR was 15% and 25%, respectively, with stable disease common (29). These results support MAPK-targeted therapy in molecularly selected pLGG, not a uniform response across PXA, ganglioglioma, epithelioid glioblastoma, and adult diffuse glioma.

Thyroid cancer without brain metastasis. Final ROAR results for 36 patients with advanced BRAF V600E ATC showed investigator-assessed ORR 56%, median PFS 6.7 months, median OS 14.5 months, and median response duration 14.4 months with dabrafenib plus trametinib (26). These are systemic ATC outcomes. The publication did not separately identify a brain-metastasis subgroup or report intracranial response, CNS PFS, or brain pharmacokinetics; ROAR therefore cannot be used as direct TCBM intracranial evidence.

Thyroid cancer brain metastases. A single-center retrospective study included 16 patients with BRAF V600E-positive thyroid cancer, comprising 10 PTC and 6 ATC cases. Reported systemic ORR was 50% in PTC and 67% in ATC; median PFS and OS were not reached in PTC and were 2.7 and 3.6 months, respectively, in ATC (30). Only two patients had baseline brain metastases. One evaluable patient had a 39.7% intracranial and 74.9% extracranial reduction after prior stereotactic radiotherapy (SRT); the second was unevaluable at cutoff and later had a 48.6% reduction. This case-level observation is exploratory. The very small denominator and prior SRT prevent attribution of intracranial change solely to dabrafenib plus trametinib and do not provide a cohort intracranial ORR.

Melanoma brain metastases as an indirect comparator. COMBI-MB reported an intracranial response rate of 58%, median PFS 5.6 months, and median OS 10.8 months in its 76-patient cohort A treated with dabrafenib plus trametinib (31). BREAK-MB reported investigator-assessed intracranial responses of 39.2% without prior local therapy and 30.8% after prior local therapy among BRAF V600E cohorts treated with dabrafenib (32). In the 24-patient vemurafenib pilot, 10 patients achieved an overall partial response involving intracranial and extracranial disease. Among 19 patients with measurable intracranial lesions, seven had >30% intracranial regression and three achieved a confirmed intracranial partial response (33). Evidence derived from melanoma brain metastases is indirect and cannot be directly generalized to thyroid cancer brain metastases or primary BRAF V600E-mutant CNS tumors.

Across these studies, the clinically actionable conclusion is narrower than a cross-tumor efficacy claim. Prospective glioma and pLGG studies show that molecular selection can identify patients with CNS tumors who may respond to MAPK inhibition. ROAR supports systemic activity in ATC. The Ando series indicates that intracranial change can be observed in a patient with TCBM, but it cannot separate drug effect from prior SRT or define response probability. Melanoma trials demonstrate that BRAFi-containing regimens can have intracranial activity in another lineage and provide design precedents for CNS endpoints; they do not supply missing TCBM outcomes. Future thyroid studies should therefore prespecify CNS eligibility, prior local treatment, lesion-level assessment, and separate systemic and intracranial endpoints.

## 7 Emerging combination strategies and evidence gaps

Combination strategies should be matched to evidence maturity. In BRAF- or NRAS-mutant melanoma models, MEK inhibition enhanced presentation of targetable major histocompatibility complex class I (MHC-I) antigens, while biopsies from patients receiving BRAF-targeted therapy showed increased melanoma-antigen expression, CD8-positive infiltration, and changes in programmed cell death protein 1/programmed death-ligand 1 (PD-1/PD-L1) markers (34, 35). These findings cannot be directly transferred to TCBM.

A peer-reviewed study in BRAF V600E-mutant glioma models provides more proximal preclinical evidence. BRAFi/MEKi induced cell-state and interferon/PD-L1-related immune changes, and concurrent immune-checkpoint inhibition improved mouse survival in a T-cell-dependent manner (36). This supports investigation in BRAF V600E glioma but is not clinical evidence. In melanoma CNS metastases, the republished phase 2 TRICOTEL study of atezolizumab, vemurafenib, and cobimetinib reported an independent-review intracranial ORR of 42%, median intracranial response duration 7.4 months, and grade 3 or worse treatment-related adverse events in 41 of 60 treated patients (68%); one treatment-related death from limbic encephalitis was reported (37). This melanoma experience remains indirect for TCBM and selected primary CNS tumors.

No clinical triplet strategy combining MAPK and immune-checkpoint inhibition has been established for TCBM or the selected primary CNS tumors. Prospective evaluation would need to address overlapping toxicities, treatment sequencing, integration with surgery or radiotherapy, steroid exposure, resistance management, and CNS-specific efficacy. Likewise, ferroptosis sensitization remains a preclinical strategy. Translation requires agents with appropriate systemic and CNS exposure, pharmacodynamic evidence of lipid-peroxidation engagement, and a therapeutic window in neural tissue.

Several priorities follow from the evidence gaps. First, paired thyroid primary and brain-metastasis samples should define BRAF status, co-alterations, differentiation state, and cell-death programs. Second, disease-specific organoid, xenograft, or immunocompetent models should test apoptosis and ferroptosis mechanisms without conflating thyroid, melanoma, and glioma lineages. Third, clinical studies should prospectively define measurable CNS disease, prior local therapy, intracranial response, CNS PFS, and systemic outcomes separately. Fourth, compound-specific unbound exposure and BTB heterogeneity should be integrated with pharmacodynamic sampling. These steps would convert an indirect cross-tumor framework into testable, disease-specific evidence.

Treatment sequencing deserves equal attention. Surgery and radiotherapy may be required for local control or urgent neurologic indications, while systemic therapy may address extracranial and intracranial disease; their contributions should not be collapsed into one response attribution. For combination trials, tolerability must be assessed in the intended CNS population rather than borrowed from a different tumor type. Longitudinal sampling should distinguish primary resistance, adaptive signaling during therapy, and acquired progression. These design features are necessary before a mechanistic combination can be promoted from rationale to disease-specific clinical strategy.

## 8 Conclusions

BRAF V600E identifies a therapeutically relevant subgroup within several primary CNS tumors and thyroid cancers, but it does not erase differences in lineage, histology, co-alterations, and the brain microenvironment. Apoptosis-related evidence is supported by components demonstrated in melanoma and extracranial thyroid-cancer models; the integrated pathway remains insufficiently validated in PXA, ganglioglioma, and TCBM. Ferroptosis sensitization is supported by preclinical BRAF V600E ATC studies outside the CNS, whereas FSP1/CoQ10 and DHODH are general mechanistic frameworks.

Clinical data support BRAFi/MEKi activity in molecularly selected glioma and systemic ATC. Direct intracranial evidence in TCBM is limited to very few, confounded observations, and ROAR systemic ATC outcomes cannot fill that gap. BBB/BTB heterogeneity, efflux, microenvironmental signaling, and prior local therapy must be measured rather than inferred from cross-trial response. Disease-specific mechanistic validation and prospective CNS-endpoint studies are therefore required before ferroptosis sensitization or immunotherapy triplets can be considered established strategies in these target settings.

For practice and research, the central discipline is to preserve the denominator and the disease context behind every statement. A systemic ATC response is not an intracranial TCBM response; a melanoma brain-metastasis result is an indirect comparator; and an extracranial cell-line mechanism is not patient-level evidence. Applying those boundaries does not diminish the relevance of BRAF V600E. Instead, it identifies where targeted therapy is supported, where a pathway provides a testable rationale, and where direct evidence is currently lacking. The next advances will depend on molecularly annotated CNS cohorts, paired tissue and pharmacokinetic analyses, and trials that report intracranial and systemic outcomes separately. Transparent reporting of prior surgery, radiotherapy, steroid use, evaluable lesions, and reasons for missing CNS assessments will be essential. These data will also clarify whether resistance reflects incomplete exposure, pathway reactivation, altered cell-death thresholds, microenvironmental adaptation, or several factors operating together in prospective disease-specific studies.
